# Effects of triamcinolone acetonide on vessels of the posterior segment of the eye

**Published:** 2009-12-08

**Authors:** Fatemeh Valamanesh, Marianne Berdugo, Florian Sennlaub, Michèle Savoldelli, Cyndie Goumeaux, Marianne Houssier, Jean-Claude Jeanny, Alicia Torriglia, Francine Behar-Cohen

**Affiliations:** 1 INSERM; UMRS 872 Physiopathology of ocular diseases: Therapeutic innovations. Centre de Recherche des Cordeliers. 15 rue de L’Ecole de Médecine 75006 Paris France; 2 Université Paris Descartes; Paris, France; 3 Université Pierre et Marie Curie, Paris, France; 4 Laboratoire d’Innovations Thérapeutiques, Fondation Rothschild, 48 rue Mathurin Moreau 75019 Paris, France; 5 Assistance Publique-Hôpitaux de Paris, Hôtel-Dieu de Paris, Department of Ophthalmology, 1 place du Parvis Notre-Dame 75001 Paris, France

## Abstract

**Purpose:**

This study investigates the effects of triamcinolone acetonide (TA) on retinal endothelial cells in vitro and explores the potential vascular toxic effect of TA injected into the vitreous cavity of rats in vivo.

**Methods:**

Subconfluent endothelial cells were treated with either 0.1 mg/ml or 1 mg/ml TA in 1% ethanol. Control cells were either untreated or exposed to 1% ethanol. Cell viability was evaluated at 24 h, 72 h, and five days using the tetrazolium 3-(4,5-dimethylthiazol-2-yl)-2,5 phenyltetrazolium bromide test (MTT) and lactate dehydrogenase (LDH) assays. Cell proliferation was evaluated by 5-bromo-2-deoxyuridine (BrdU) test. Apoptosis was evaluated by terminal deoxynucleotidyl transferase dUTP nick end labeling assay (TUNEL assay), annexin-binding, and caspase 3 activation. Caspase–independent cell deaths were investigated by immunohistochemistry using antibodies against apoptosis inducing factor (AIF), cytochrome C, microtubule-associated protein (MAP)-light chain 3 (MAP-LC3), and Leukocyte Elastase Inhibitor/Leukocyte Elastase Inhibitor-derived DNase II (LEI/L-DNase II). In vivo, semithin and ultrathin structure analysis and vascular casts were performed to examine TA-induced changes of the choroidal vasculature. In addition, outer segments phagocytosis assay on primary retinal pigment epithelium (RPE) cells was performed to assess cyclooxygenase (*COX-2*) and vascular endothelial growth factor (*VEGF*) mRNAs upregulation with or without TA.

**Results:**

The inhibitory effect of TA on cell proliferation could not explain the significant reduction in cell viability. Indeed, TA induced a time-dependent reduction of bovine retinal endothelial cells viability. Annexin-binding positive cells were observed. Cytochrome C was not released from mitochondria. L-DNase II was found translocated to the nucleus, meaning that LEI was changed into L-DNase II. AIF was found nuclearized in some cells. LC3 labeling showed the absence of autophagic vesicles. No autophagy or caspase dependent apoptosis was identified. At 1 mg/ml TA induced necrosis while exposure to lower concentrations for 3 to 5 days induced caspase independent apoptosis involving AIF and LEI/L-DNase II. In vivo, semithin and ultrathin structure analysis and vascular casts revealed that TA mostly affected the choroidal vasculature with a reduction of choroidal thickness and increased the avascular areas of the choriocapillaries. Experiments performed on primary RPE cells showed that TA downregulates the basal expression of *COX-2* and *VEGF* and inhibits the outer segments (OS)-dependent COX-2 induction but not the OS-dependent VEGF induction.

**Conclusions:**

This study demonstrates for the first time that glucocorticoids exert direct toxic effect on endothelial cells through caspase-independent cell death mechanisms. The choroidal changes observed after TA intravitreous injection may have important implications regarding the safety profile of TA use in human eyes.

## Introduction

Glucocorticoids are commonly used in the treatment of ocular pathologies associated with vascular leakage, vessels abnormalities, and ocular neovascularization. Intravitreous injections of Triamcinolone Acetonide (TA) are currently used for the treatment of macular edema [1,2]. They induce a dramatic reduction of macular thickness but this is inconsistently correlated with long-term functional recovery. When used for the treatment of choroidal neovascularizations associated with Age-related Macular Degeneration (AMD) or other ocular neovascularisations, TA decreases the vascular leakage on the short-term [3]. However, its long-term effect on visual acuity remains controversial [4-6]. For the treatment of retinal neovascularization, TA has been used mostly in combination with other antivascular strategies [7,8]. On capillary eyelid hemangiomas, glucocorticoids seem to have beneficial effects through a reduction in the size of the vascular lesion [9].

The vascular effects of TA have been confirmed in animal models of choroidal (CNV) and retinal neovascularization. In the rat model of laser-induced CNV, TA not only decreased fluorescein angiography (FA) leakage [10,11], but it also reduced the CNV membrane diameter and thickness [11,12], and transiently inhibited CNV formation [13]. In the rat model of retinopathy of prematurity, TA reduced the retinal neovascularization in a dose-dependent manner [14-17] and reduced the vascular leakage [16].

In vitro, TA has been shown to downregulate the expression of tumor necrosis factor α, vascular endothelial growth factor (VEGF), interleukin 1, and matrix metalloproteinases, and subsequently influence neovascularization [16]. TA has also been reported to have direct effects on endothelial cells through an antiproliferative effect [17] and a reduction in the interferon-induced permeability of human choroidal endothelial cells [18].

Beside their known effects on vascular permeability, the in vivo antiangiogenic/angiostatic activity of corticosteroids results from many factors, including their anti-inflammatory activities. However, it remains unknown if TA can directly induce vascular endothelial cell death. In this paper, we investigated the mechanisms of the effects of TA on retinal endothelial cells in vitro and in the rat retinal and choroidal vascularization in vivo.

## Methods

### Reagents

All culture reagents were obtained from Gibco (New York, NY). Corticosteroids were purchased from Sigma (Saint-Quentin-Fallavier, France) except for Kenacort Retard, which was purchased from Bristol-Myers Squibb (Paris, France).

### Culture of bovine retinal endothelial cells and treatments

Bovine retinal endothelial cells (BRECs) were seeded in Dulbecco’s modified Minimal Essential Medium (DMEM)/glutamax supplemented with 10% calf serum and 20 ng of VEGF (Sigma, St. Louis, MO), at 37 °C in a humidified atmosphere containing 5% CO_2_. After 24 h the cells were treated for either 24, 72 h, or 5 days with 0.1 or 1 mg/ml TA that had been dissolved in 1% ethanol (TA-E; 99.9% ethyl alcohol; VWR, Paris, France). Control cells were either not treated or treated with 1% ethanol in medium. Before treatment, cells were maintained in medium supplemented with 2% of fetal calf serum (FCS) for 24 h. For treatment, cells were cultured in medium supplemented with 2% FCS without VEGF. In some experiments, cells were treated after confluence was reached.

### Analysis of TA effects on cultured cells

#### Cell viability evaluation using MTT test

Viability was assessed by the tetrazolium 3-(4,5-dimethylthiazol-2-yl)-2,5 phenyltetrazolium bromide test (MTT; Sigma Chemical) using 2.10^5^ cells per well in 24-well plates. Briefly, culture medium was removed and 250 µl of MTT (1 mg/ml in 1× PBS: 137 mM NaCl, 2.7 mM KCl, 10 mM Na_2_HPO_4_, 2 mM KH_2_PO_4_, pH 7.4) were added to each well. The wells were then incubated for 1 h at 37 °C, lysed with 250 µl of isopropanol, and assessed by measuring absorption at 570 nm versus 630 nm using a microplate reader (BioRad, San Diego, CA). Using a standard curve for each experiment, we correlated the color intensity observed in each well to the number of viable cells assessed by the trypan blue exclusion assay.

#### Evaluation of TA-induced viability reduction: lactate dehydrogenase cytotoxicity assay

In 24-well plates, 2.10^5^ BRECs/well were treated with TA-E for 24 h, 72 h, or five days. Released lactate dehydrogenase (LDH) in culture supernatants was measured with a cytotoxicity detection kit (Roche Diagnostics, Mannheim, Germany). Dye absorbance was measured at 490 nm versus 630 nm using a standard microplate reader. Positive control was obtained by the complete lysis of control wells with 0.2% Triton X100. Because treatment of BRECs with TA-E at 1 mg/ml induced mostly necrosis, resulting from the direct contact of the unsolved crystals with the cells, all further analysis were only performed with TA-E at 0.1 mg/ml.

### Evaluation of cell proliferation measuring DNA synthesis rate

For proliferation assay, TA was used at 0.1 mg/ml and 0.5 μg/ml. BRECs (10^5^ cells/well) were seeded in 24-well cell culture plates and allowed to attach for 24 h. Thereafter, the cells were incubated with TA-E for 24 h and 5-bromo-2-deoxyuridine (BrdU) was added into the medium, for a final concentration of 10 µM, and allowed to incubate for 18 h. The incorporation of BrdU into the genomic DNA was determined using the ELISA BrdU labeling and detection Kit III (Roche Molecular Biochemicals, Germany).

For other experiments, BRECs were seeded at 5x10^4^ cells/well in the Lab-Tek chambered Coverglass and grown at 37 °C in a humidified atmosphere containing 5% CO_2_ and 95% air for 24 h. They were then treated with 0.1 mg/ml TA-E, which is a concentration that allows further analysis. Control cells were run using the culture medium alone or the culture medium containing 1% ethanol. All experiments were performed in triplicates and repeated twice. The Lab-Tek slides were mounted in Gel Mount (Biomeda, Burlingame, CA) before they were examined and photographed using an Olympus IX70 fluorescent microscope coupled to a digital camera. The following excitation/emission wavelengths were used: 365–420 nm; 470/510 nm; 510/560 nm.

### Detection of apoptosis using terminal deoxynucleotidyl transferase dUTP nick end labeling assay

After treatment with 0.1 mg/ml TA-E for 24 h, 72 h, or five days, the cells were rinsed, air-dried, and fixed with 4% paraformaldehyde (Merck Eurolab, Fontenay sous-bois, France) one hour at room temperature. The cells were rinsed during 5 min in PBS, and permeabilized with 0.3% Triton X-100 in PBS for 20 min. Cells were incubated for 30 min with 50 µl of terminal deoxynucleotidyl transferase dUTP nick end labeling assay (TUNEL; Roche Diagnostics, Mannheim, Germany) reaction mixture at 37 °C. The cells were then rinsed once with PBS, stained with DAPI (Sigma), and rinsed with PBS five times for 5 min each time. Positive controls were obtained by inducing apoptosis with 1 µM staurosporine.

### Annexin V binding

Annexin V binding was assayed with the Annexin V Apoptosis detection kit (Santa Cruz Biotechnology Inc., Santa Cruz, CA) according to the manufacturer instructions. Briefly, after treatment cells were rinsed with PBS, washed once for 5 min with 500 µl per well of assay buffer and incubated 15 min at room temperature with 2 µg/ml of Fluorescein isothiocyanate (FITC)–labeled Annexin V. Cells were then, rinsed with PBS, fixed with 4% paraformaldehyde, rinsed with PBS, stained with DAPI and rinsed five more times for 5 min with PBS.

### Western blot analysis

Total protein extracts were obtained by lysis of BRECs in Laemmli sample buffer. Extracted proteins were separated by SDS–PAGE, immobilized on nitrocellulose membrane (Millipore, Billerica, MA) and blotted with affinity purified rabbit anti-active Caspase-3 polyclonal antibody in a 1:500 dilution (Calbiochem, San Diego, CA). The secondary goat anti-rabbit IgG (Vector Laboratories, Burlingame, CA) was used in a 1:5,000 dilution. The amount of total protein extracts analyzed was 30 μg/lane. Positive controls for caspase-3 activation were run on HL-60 cells, cultured in RPMI 1640 supplemented with 10%. Fetal calf serum, treated for 24 h with 50 μM etoposide [19].

### Immunocytochemistry

After the different treatments BREC cells were stained with the following antibodies: anti-cytochrome C [20] (Sigma, Steinheim, Germany) diluted 1:250 in PBS containing 1% BSA (BSA 98%; Sigma, Steinheim, Germany); anti-apoptosis-inducing factor (AIF) [21] (Sigma, Saint-Quentin Fallavier, France) diluted 1:100 in PBS containing 1% BSA; anti-microtubules-associated protein and light chain 3 (MAP-LC3) [22] (Santa Cruz Biotechnology) diluted 1:50 in PBS containing 1% fat-free milk; and polyclonal anti- L -DNase II antibody [23,24] diluted 1:100 in PBS containing 1% skimmed milk. After incubation with the specific primary antibodies, the cells were rinsed three times for 5 min with PBS, and incubated for 1 h with goat Alexa Fluor anti-rabbit IgG (Molecular Probe Invitrogen, NewYork, NY), or rabbit anti goat Texas red dye-conjugated (Jackson Immuno Research Laboratories Inc., Suffolk, UK) diluted 1:250 in PBS containing 1% BSA. PBS containing 1% BSA or 1% skimmed milk was used instead of the primary antibody as negative controls.

For autophagic positive controls, BRECs were cultured for 24 h in amino acid-depleted medium, e.g Hanks Buffered Salt Saline (HBSS, Gibco, New York, NY) complemented with 20 mM glucose [25]. Slides were mounted in Gel Mount and examined with a fluorescent microscope Olympus IX70 coupled to a digital camera. The following excitation/emission wavelengths were used: 365–420 nm; 470/510 nm; 510/560 nm.

### Semithin and ultrathin structure analysis

Lewis rats used in this study were obtained from Roger Janvier, Le Genest Saint Isle, France. The use of animals adhered to the ARVO statement for Ophthalmic and Vision Research and protocols were approved by the ethical committee of René Descartes University of Paris. Lewis rats (6-week-old, weighing 150 g) were intravitreally injected with either 10 μl of PBS (n=3) or with 10 μl of PBS containing 40 μg of TA (n=3). Thirty days after injection, the rats were euthanized with a CO_2_ overdose, and their eyes processed for semithin and transmission electron microscopy analysis. The eyes were fixed in 2.5% glutaraldehyde cacodylate buffer (Na 0.1 M, pH 7.4) for 1 h. They were then dissected, and the posterior segments were fixed for 3 h. Specimens were fixed in 1% osmium tetroxyde in cacodylate buffer (Na 0.1 M, pH 7.4) and progressively dehydrated in graduated ethanol solution (50, 70, 95, and 100%). Each posterior segment was separated in two samples, included in epoxy resin and oriented. Sections were cut to 1 µm thickness with an ultra microtome Reichert Ultracut (Leica, Biel, Switzerland) and stained with toluidine blue. Sections cut to 80 nm thick were stained by uranyl acetate and lead citrate, and analyzed with a Philips CM10 electron microscope.

### Vascular corrosion casts

Lewis rats (12, 8-week-old, weighing 150 g) were intravitreally injected with either 10 µl of PBS (n=6) or with 10 µl PBS containing 40 µg of TA (n=6). After 30 days the animals were sacrificed by CO_2_ inhalation followed by a thoracotomy. A catheter was introduced into the aorta through the left heart ventricle, and the right auricle was cut to allow evacuation of injected products. A perfusion was performed with a mixture of red Mercox resin and catalyst (Ladd Research, Williston, VT). Eyes were extracted and lenses were removed. Tissues were conserved overnight at 37 °C in PBS to allow complete polymerization, and then digested by 5% KOH for two weeks at 37 °C until only the vascular corrosion casts remained. Distilled water was used to remove salt, and the mold was dried. Only corrosion casts with completely filled iris vessels were used. This was to exclude corrosion casts from incomplete perfusion. Retinal vasculature was removed with forceps. The specimens were mounted on Stretched Specimen Mount (SSM) stubs, coated with gold palladium, and scanned at an accelerating voltage of 117 kV. To measure the thickness of the choriocapillary lumen, we cut corrosion casts paracentrally (1 mm from the aperture of the optic nerve) and positioned for perpendicular views of the choriocapillaries. To analyze the intercapillary space (avascular area), we positioned the casts for frontal views of the choriocapillaries. Electron micrographs were scanned and analyzed using Image J Software. The avascular areas were measured on frontal views and expressed as the percentage of intercapillary surface (space between the plastic capillary casts) of the whole area (n=6 rats; five pictures/cast).

### Effect of TA on VEGF and COX-2 expression in primary retinal pigment epithelium (RPE) cells

#### Rat RPE primary culture

Ten-day-old Wistar rat pups were sacrificed by CO_2_ inhalation and the eyes were enucleated. Eyes were maintained at room temperature overnight in DMEM (Invitrogen, Cergy Pontoise, France) then incubated 45 min with 2 mg/ml trypsin/collagenase I at 37 °C. After trypsin inhibition with DMEM containing 10% FCS, the RPE layer was harvested. The RPE was plated in 12-well plates at a rate of one RPE from one eye per well in DMEM containing 10% FCS, 1% penicillin/streptomycin, and 0.2% fungizone. Cells were maintained for 12 days before the phagocytosis assay [26].

### Outer segments isolation and phagocytosis assay

Outer segments (OS) were isolated following established protocols [27]. Briefly, 20 pig retinas were dissected and homogenized in 20% sucrose buffer, 20 mM Tris, 2 mM MgCl_2_, and 0.13 mM NaCl (pH 7.2). Retina samples were centrifuged on a sucrose gradient (50%, 27%) 1h at 100,000× g, and OS were harvested at the ring interface and diluted in DMEM. Centrifugation for 10 min at 10,000× g was performed, and the pellet was resuspended in DMEM to obtain a stock solution of 10^8^ OS/ml.

Before treatment, RPE cells were maintained in medium supplemented with 2% of FCS for 24 h. Confluent rat primary RPE cells were incubated with 0.1 mg/ml TA-E. Control cells were run using the culture medium alone. After 24 h of treatment, the cells were rinsed once with PBS and incubated with 150 μl of 10^8^ outer segments/ml. After 1 h, 850 µl of complete medium was added. After 6 h, cells were washed and 350 μl of guanidine thiocyanate lysis buffer (RNeasy mini kit; Qiagen, Courtaboeuf, France) was added for RNA extraction.

### Reverse transcription and real-time polymerase chain reaction

Total RNA was isolated with RNeasy plus Mini Kit (Qiagen, Courtaboeuf, France). Single-stranded cDNA was synthesized from total RNA using random primer and superscript reverse transcriptase (Invitrogen). Subsequent real-time polymerase chain reaction (RT–PCR) was performed using cDNA, qPCR SuperMix-UDG Platinum SYBR Green (Invitrogen), and the and 0.5 pmol/µl of the primers described on Table 1.

**Table 1:** primers used in qPCR.

**Name**	**Sequence (5′-3′)**
18S sense	TGCAATTATTCCCCATGAACG
18S antisense	GCTTATGACCCGCACTTACTGG
VEGF sense	TGGGATGGTCCTTGCCTC
VEGF antisense	TCGCTGGAGTACACG GTGGT
COX2 sense	TGCTACCATCTGGCTTCGGGAG
COX2 antisense	ACCCCTCAGGTGTTGCACGT

PCR reactions were performed in 40 cycles of 15 s at 95 °C, 45 s at 60 °C. No product was obtained in control reactions where reverse transcriptase was omitted.

### Statistical analysis

Data are represented as mean±standard error of the mean (SEM) and compared using the nonparametric Mann–Whitney U test. P values were calculated for a confidence interval of 95% and p values of less than 0.05 were considered significant.

## Results

### Effect of TA on BREC viability

**Figure 1:**
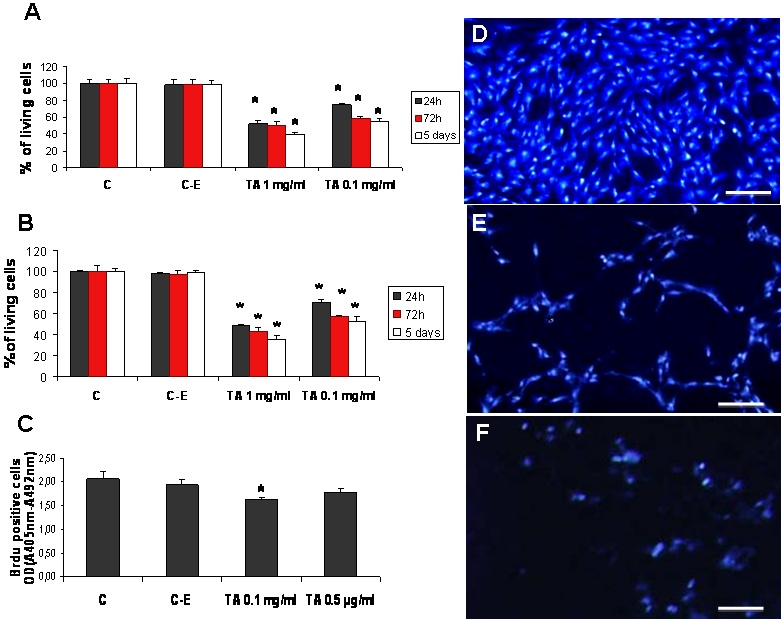
Effect of triamcinolone acetonide on BRECs viability and proliferation. Cell viability was evaluated using an MTT assay. Subconfluent (**A**) or confluent BRECs (**B**) were exposed to 0.1 or 1 mg/ml TA in 1% ethanol for 24 h (dark-gray columns), 72 h (red columns), or five days (white columns). Control cells were either exposed to 1% ethanol (**C-E**) or were left untreated (**C**). **C**: Cell proliferation was evaluated using BrdU labeling detection after 24 h of treatment with 0.1 mg/ml or 0.5 µg/ml TA. Control cells were either left untreated (**C**) or were treated with 1% ethanol (**C**-**E**). Results are expressed as mean±standard error; with *p<0.05 for all columns versus control. Four animals were used in each experiment. DAPI staining of untreated control cells are shown in (**D**), 0.1 mg/ml TA-treated cells in (**E**) and 1 mg/ml TA-treated cells in (**F**), all of them after five days of treatment. Scale bar represents 50 µm.

The percentage of living cells was reduced to 75±1.1%, p<0.05 after exposure to 0.1 mg/ml of TA for 24 h. This survival was reduce to 52±2.9% p<0.05 as concentration of TA increased to 1 mg/ml. The rate of survival was further reduced as exposure time increased, being of 58±2.3% and 50±3.7% for 0.1 mg/ml, and 1mg/ml of TA, respectively, after 72 h and of 55±2% and 39±0.9% p<0.05, respectively, for the same concentrations after 5 days of treatment. TA therefore decreased the number of living cells as compared to control cells, with an increased effect upon exposure duration. Photomicrographs of DAPI-stained BREC nuclei in culture showed that after 72 h (not shown) or five days (Figure 1D-F) of treatment with 0.1 mg/ml TA-E, the cells were not only reduced in number (Figure 1D), but also organized in a ramified manner (Figure 1E). After treatment with 1 mg/ml for 72 h or five days (Figure 1F), the number of cells was reduced and many cellular debris were present in the culture medium. To evaluate whether the reduction in the number of living cells resulted from a reduction in proliferation or in TA-induced cell toxicity, we performed the same experiments on confluent cells. As shown on Figure 1B, TA induced a similar reduction in the number of living cells, this effect increased upon the duration of exposure and the concentration, suggesting that the reduction in the number of living cells did not result solely from an inhibition of cell proliferation.

### Inhibition of BREC proliferation

To further characterize the effect of TA on endothelial cell proliferation, we evaluated the DNA synthesis rate using the BrdU test. As shown in Figure 1C, 0.1 mg/ml TA-E significantly reduced the proliferation rate of BRECs from OD=2.06±0.09 in control cells to OD=1.62±0.02, n=4, *p<0.05 in TA-E treated cells. This effect was not observed with a lower concentration of TA-E: 0.5 µg/ml (OD=1.8±0.03).

### Evaluation of retinal endothelial cell death after exposure to TA

#### Necrosis in BREC exposed to TA: Measurement of LDH release

The reduction in the number of living cells could not be explained only by a decrease of proliferation. Therefore, cell death mechanisms were explored. We first measured the amount of free LDH as a marker of passive cell death (e.g., necrosis; Table 2). LDH is a cytoplasmic enzyme not released during active cell death like apoptosis, paraptosis, or autophagy, but released in necrosis due to the early increase of plasma membrane permeabilization. No necrosis was observed in control cells; exposure of BRECs to 1 mg/ml TA-E induced necrosis in 23.7% of the cells after 24 h of exposure, in 23.4% after 72 h, and in 24.3% of the cells after five days (p<0.05). After exposure to 0.1 mg/ml TA-E, a significantly lower release of LDH than before was observed, reaching 3.6% after 24 h, 4.2% after 72 h and 5.1% after five days of treatment (p<0.05). Taking into account the inhibition of proliferation and the percentage of necrosis occurring in 0.1 mg/ml TA-E treated cells; it is possible there is another type of cell death that could possibly explain the reduced number of living cells after 72 h and five days.

**Table 2:** LDH release

**Treatment**	**% of cytotoxicity**		**% of cytotoxicity**		**% of cytotoxicity**	
	**24 h**	**p**	**72 h**	**p**	**5 days**	**p**
TA 1 mg/ml	23.7	<0.05	23.4	<0.05	24.3	<0.05
TA 0.1 mg/ml	3.6	<0.05	4.24	<0.05	5.1	<0.05
C-E	0		0		0	
C	0		0		0	
Triton 0.2%	100		100		100	

Measure of LDH release in BRECs after 24 h, 72 h, and 5 days of treatment with 1 mg/ml or 0.1 mg/ml of TA. Control cells were either not treated (**C**), 1% ethanol-treated (**C-E**), or 0.2% triton-treated. No LDH release was found in control cells and a small increase is seen in cells treated with 0.1mg/ml of TA. The use of 1 mg/ml of TA drastically increase LDH release.

When 1 mg/ml TA was dissolved in 1% ethanol, crystals remained undissolved and stayed adherent to the cell membranes, resulting in the direct cytotoxic observed effects. Therefore, for further analysis, only cells treated with 0.1 mg/ml TA-E were used. Since the TA effect on cell viability was increased with the time of treatment, cell death was analyzed after 72 h and five days of exposure.

### Caspase-dependent apoptosis

**Figure 2:**
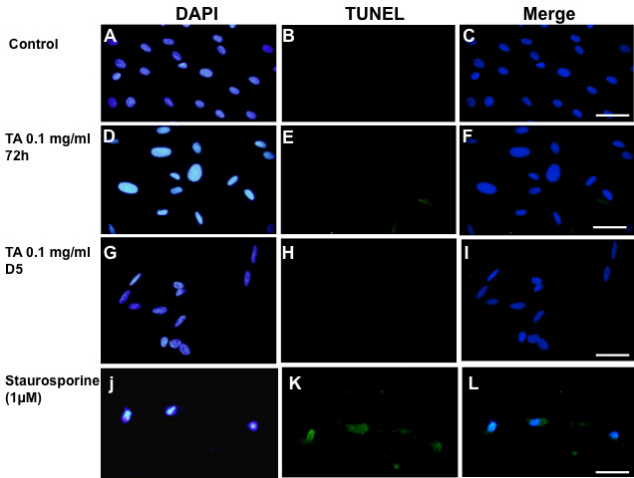
Absence of TUNEL-positive apoptosis in BRECs treated with triamcinolone acetonide. Control cells were treated with the TA vehicle,1% ethanol, (**A**-**C**). No TUNEL-positive cells could be observed after exposure to 0.1 mg/ml TA for 72 h (**D**-**F**) or five days (D5, **G**-**I**). As a positive control for the TUNEL technique, BRECs were treated with 1 μM staurosporine (**J**-**L**). Nuclei were stained with DAPI (**A**, **D**, **G**, **J**). Scale bar represents 60 µm.

Caspase-dependent apoptosis was evaluated by TUNEL assay, annexin-V binding, caspase 3 activation, and cytochrome C release. As shown in Figure 2, no TUNEL-positive cells were detected after treatment with 1% ethanol (Figure 2A-C). In addition, no TUNEL-positive cells could be detected in cells treated with 0.1 mg/ml TA-E after 72 h (Figure 2D-F) or even after five days of exposure (Figure 2G-I). However, when cells were exposed to 1 µM staurosporine, a known inducer of caspase-dependent cell death, TUNEL-positive cells with condensed nuclei were observed (Figure 2J-L).

**Figure 3:**
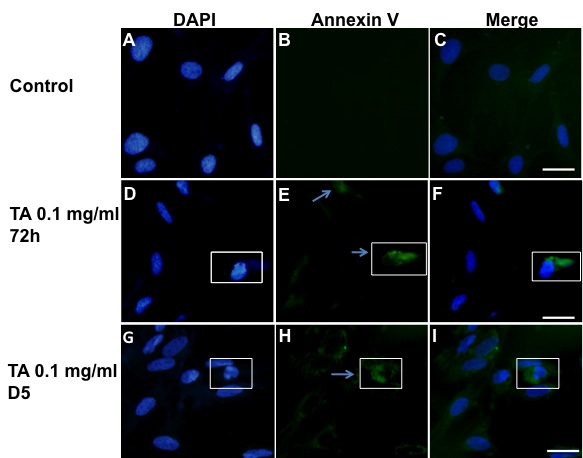
Annexin V binding of TA treated BRECs. Annexin-V binding was performed in control cells, treated with 1% ethanol (the TA vehicle; **A**-**C**) or with 0.1% TA for 72 h (**D**-**F**) or five days (**G**-**I**). Condensed nuclei are seen in TA treated cells (**D** and **G**, squares) as well as Annexin-V positive cells (**E** and **H**, squares). Arrows indicates Annexin-V positive cells. Scale bar represents 50 µm.

During the early phases of any type of apoptosis, phosphatidyl serine translocates to the external surface membrane of the cell [28] and binds annexin-V [29]. As shown in Figure 3, while no annexin V was found to bind on the surface of control cells (Figure 3A-C), positive, stained cells were detected after 72 h and five days of exposure with 0.1 mg/ml TA-E (Figure 3D-F,G-I). After 72 h of exposure to TA, annexin-V positive cells displayed condensed or fragmented nuclei (Figure 3D-F insets), while after five days some cells displaying normal (uncondensed) nuclei also started annexin-V binding (Figure 3G-I insets).

**Figure 4:**
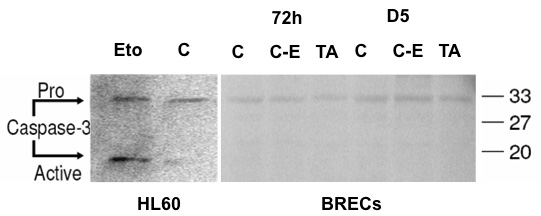
Caspase 3 western-blot analysis. As a positive control HL-60 cells were treated with etoposide (Eto). The cleaved, active form of caspase 3 is seen. Expression of pro-caspase 3 is seen en BRECs in all conditions. No expression of activated caspase 3 was detected in any of the treated BRECs, demonstrating that triamcinolone acetonide (TA) did not induce caspase 3 activation.

The results discussed in the previous section suggested the activation of apoptosis with no activation of caspase. To assess this point, we performed western blot analysis of caspase-3 activation. The results obtained revealed the presence of pro-caspase-3 and the absence of activated caspase-3 in BRECs treated during 72 h or five days with either nothing, 1% ethanol medium, or in 0.1 mg/ml TA (Figure 4). In positive control HL-60 cells treated with etoposide, pro-caspase-3 as well as activated caspase-3 were detected (Figure 4). These experiments showed that a noncaspase dependent form of apoptosis could intervene.

**Figure 5:**
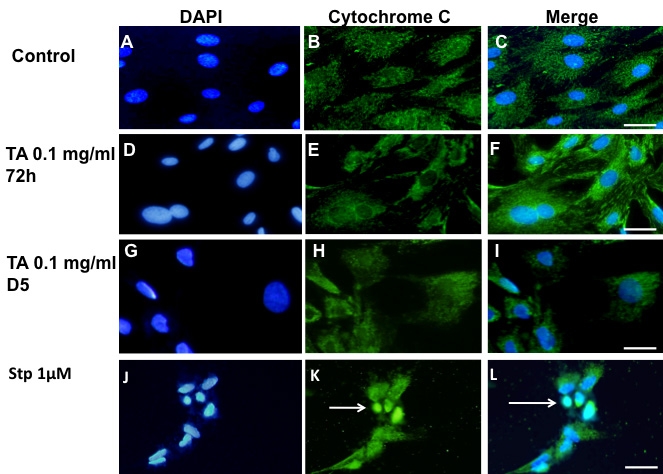
Cytochrome C release in TA treated BRECs. Absence of cytochrome C release in triamcinolone acetonide (TA)-treated BRECs. Control cells are shown in **A** to **C**. TA treated cells for 72 h are shown in **D** to **F** and for 5 days in **G** to **I**. No cytochrome C release is seen in these cells. Compare to cells treated with staurosporin (Stp) used as positive control (**J**-**L**, arrow in **K** and **L**). Nuclei were stained with DAPI (**A**, **D**, **G**, **J**). Scale bar represents 50 µm.

After a toxic stress caspase 3 is, in most of the cases, activated though the intrinsic pathway, in which, the release of cytochorme C from the mitochondria is a key event. We investigated then if cytochrome C was released. In BRECs treated during 72 h or five days with 0.1 mg/ml of TA-E (Figure 5D-F,G-I), no release of cytochrome C from mitochondria into the cytoplasm could be observed. The fluorescent labeling appeared indeed located in mitochondria, and no diffuse or nuclear fluorescence was present, indicating that mitochondria have not released cytochrome C. In contrast, in BRECs treated with staurosporine, cytochrome C was located in condensed nuclei of apoptotic cells (Figure 5 J-L, arrows).

### Caspase-independent apoptosis in triamcinolone acetonide-induced toxicity

As the cell death induced by TA seemed to be caspase independent, we analyzed two markers of caspase-independent apoptosis in TA-treated BRECs: leukocyte elastase inhibitor/ L-DNase II (LEI/L-DNase II) and apoptosis inducing factor (AIF).

**Figure 6:**
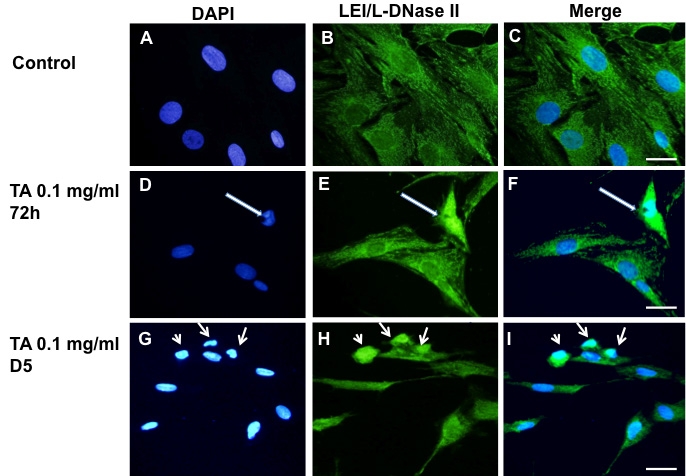
L-DNase II activation in TA treated BRECs. LEI/L-DNase II immuno-labeling was performed in control cells treated with the TA vehicle (1% ethanol). **A**-**C**: A cytoplasmic labeling is seen. In cells treated with 0.1 mg/ml TA for 72h (**D**-**F**) or 5 days (**G**-**I**), cells with condensed nuclei (arrows) present a nuclear staining for LEI/L-DNase II, indicating L-DNase II activation. Nuclei were stained with DAPI (**A**, **D**, **G**). Scale bar represents 50 µm.

LEI and L-DNase II are two forms of one protein that are recognized by the same antibody. However, while LEI is a cytoplasmic anti-protease, its nuclear translocation parallels its change into L-DNase II activity [30]. While LEI/L-DNase II fluorescent labeling was present in the cytoplasm of the control cells (Figure 6A-C), surrounding non-fluorescent nuclear shapes, a clear nuclear staining was observed in cells treated with 0.1 mg/ml TA for 72 h or for five days (Figure 6D-F,G-I, arrows). Nuclei positive for L-DNase II were condensed or fragmented, indicating a caspase-independent apoptotic pathway, taking place in BRECs cells treated with TA.

**Figure 7:**
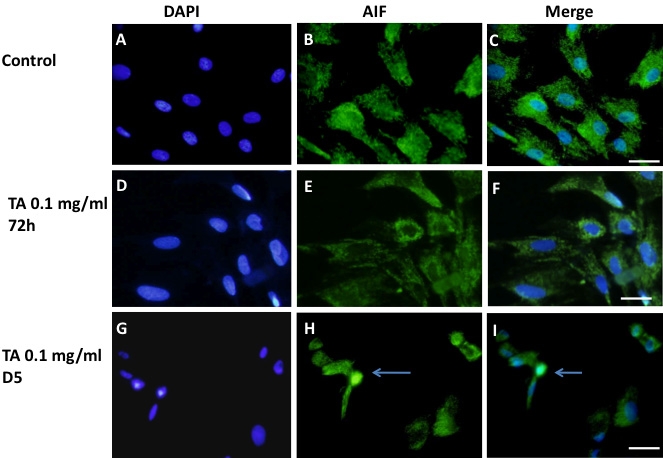
Apoptosis Inducing Factor localization in triamcinolone acetonide treated BRECs. Apoptosis inducing factor (AIF) immuno-labeling was performed in control cells treated with the TA vehicle (1% ethanol; **A**-**C**). A cytoplasmic labeling is seen. This is also the case in cells treated with 0.1 mg/ml TA for 72h (**D**-**F**). In cells treated with TA for 5 days (**G**-**I**), cells with condensed nuclei (arrows in **H** and **I**) present a nuclear staining for AIF. Nuclei were stained with DAPI (**A**, **D**, **G**). Scale bar represents 50 µm.

In control cells (Figure 7A-C), anti-AIF showed a cytoplasmic labeling; corresponding to the normal localization of AIF at the mitochondria. After 72 h of TA-E treatment no change in AIF localization was seen (Figure 7D-F). However, after exposure to 0.1 mg/ml TA-E for 5 days, some cells with condensed or fragmented nuclei showed positive nuclear staining. This suggested that caspase-independent apoptotic pathways were activated in TA-treated cells (Figure 7G-I, arrows).

### Autophagy

**Figure 8:**
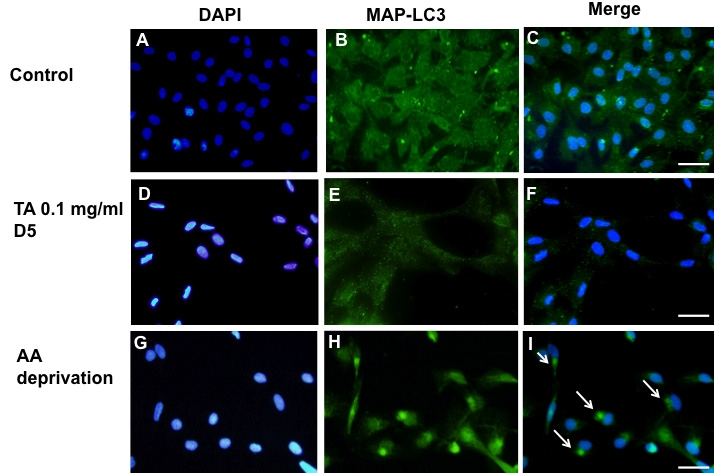
Evaluation of autophagy by microtubules-associated protein light chain 3 staining. No intracytoplasmic vacuoles were stained with anti-microtubules-associated protein light chain 3 (MAP-LC3) in control BRECs (**A**-**C**). This is also the case for BRECs exposed to 0.1 mg/ml TA for five days (**D**-**F**). In the amino acid (AA) deprived medium (positive control), BRECs show multiple MAP-LC3 positive cytoplasmic vacuoles (**G-I**, arrows in **I**). Nuclei were stained with DAPI (**A**, **D**, **G**). Scale bar represents 50 µm.

After activation of autophagy, MAP-LC3 concentrated in autophagic vesicles, giving a dotted pattern to the cells. After 72 h (not shown) or five days of treatment with 0.1 mg/ml TA-E (Figure 8D-F), no MAP-LC3-positive vesicles could be observed—meaning that autophagy was not taking place under these conditions. In cells cultured in amino acid-deprived medium, a condition known to induce authophagy, cells showed large intracytoplasmic vesicles that stained positive with anti-MAP-LC3 antibody (Figure 8G-I).

### Structural analysis of rat retinal and choroidal vasculature, one month after TA exposure

**Figure 9:**
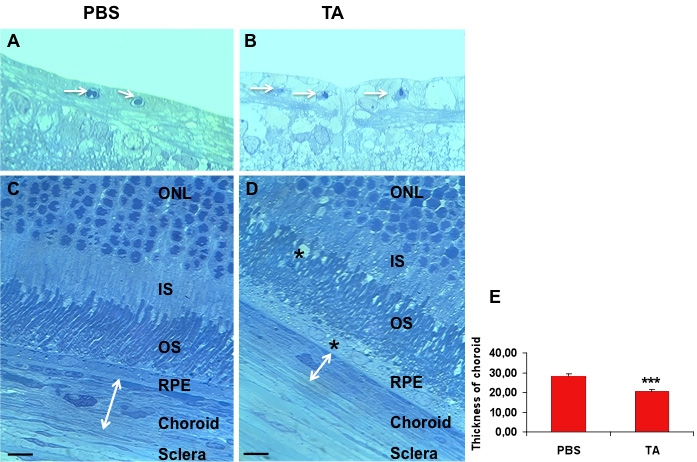
Semithin analysis of the retina-choroid. At 30 days after triamcinolone acetonide (TA) injection, the diameter of retinal vessels was reduced as compared to control PBS injected eyes (**B, A**, respectively, arrows). The thickness of the choroids was significantly reduced in TA-treated (**D**) as compared to the control eye (**C** and **E**). Vacuoles were observed in retinal pigment epithelial (RPE) cells and outer segment in the TA-treated eyes (**D**, stars). Results are expressed as mean±standard error, with ***p<0.001. Fours animals were used in this study. Abbreviations: outer nuclear layer (ONL), inner segment (IS), outer segment (OS). Scale bar represents 20 µm.

As demonstrated, the results obtained indicated that TA could affect endothelial cell viability. We investigated this hypothesis in vivo. Semithin analysis of the retina did not show evidence of alteration of the retinal vessels or capillaries, but did show that a reduction of the diameter of the superficial capillaries was evident on sections of TA-treated retinas (Figure 9B) versus control ones (Figure 9A). More striking was the significant thinning of the choriocapillaries and choroid in the TA-treated rats (Figure 9D) as compared to PBS-injected rats (Figure 9 C and E). Moreover, as previously described [26] in TA-treated rats, there were alterations of the outer segments and vacuolization of the RPE cells (Figure 9D stars).

### Analysis of rat choroidal vascular corrosion casts one month after TA exposure

**Figure 10:**
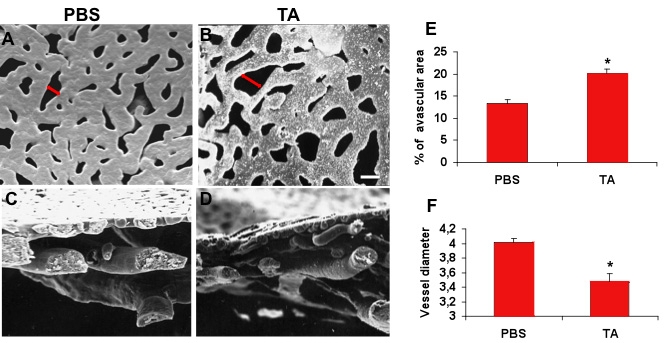
Choroidal vascular casts. Frontal view of choroidal casts showed that 30 days after injection of triamcinolone acetonide (TA), the avascular areas were increased (**B**, red double-headed arrow) as compared to the control PBS-treated eyes (**A**). Cross-sectional cuts of pericentral choroidal corrosion cast (**C**, **D**) showed a reduced thickness of the choriocapillaries in TA-treated eyes. Scale bar represents 10 µm. **E**: Quantification of the avascular areas in the choriocapillaries confirmed a significant increase in TA-treated eyes, and (**F**) mean vessel diameter was significantly reduced. Results are expressed as mean±standard error with *p<0.05 when consideringTA versus PBS. Six animals were used in this study.

To better analyze and quantify the modifications observed in the choroidal vascular network, we performed resin casts. In rats exposed to TA for a month, the casts showed that the spaces between choriocapillaries were increased and that capillary diameter was reduced as compared to PBS-injected rats. Figure 10A,B presents frontal views of choriocapillary casts of a PBS-treated rat and a TA-treated rat. Figure 10C,D shows cross-sectional cuts of pericentral choroidal corrosion casts of a PBS-treated rat and a TA-treated rat. In the TA-treated rat eyes, the avascular areas between choriocapillaries were significantly increased (n=6, *p<0.05 versus PBS) and the choriocapillary diameter significantly reduced (n=6, *p<0.05 versus PBS; Figure 10E,F).

### Modulation of VEGF and COX-2 expression in RPE cells exposed to OS phagocytosis

To explore a potential indirect mechanism responsible for TA-induced choriocapillaries alterations, we evaluated whether the expression of cyclo-oxygenase-2 (COX-2) and VEGF in RPE cells submitted to OS phagocytosis was modulated by TA exposure. We have previously shown that COX-2 knockout mice develop significant choriocapillary involution [26]. So we hypothesed that beside a direct toxic effect of TA, COX-2 downregulation could also be induced by TA on the choriocapillary in vivo.

**Figure 11:**
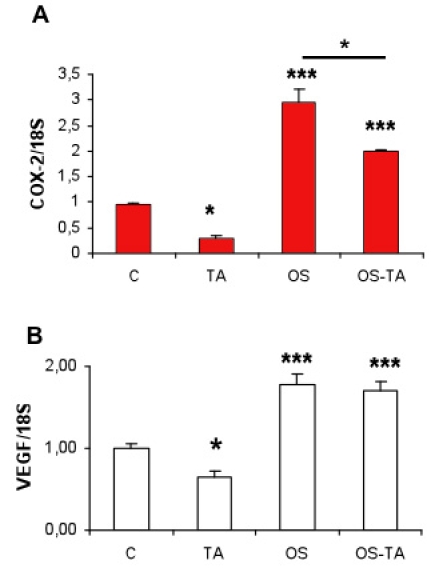
*COX-2* and *VEGF* mRNA expression in RPE cells treated with TA. *COX-2* (**A**) and *VEGF* (**B**) mRNA expression (real time quantitative PCR) in untreated control primary rat RPE cells (**C**), or in RPE cells exposed to triamcinolone acetonide (TA), outer segments (OS) or both (OS-TA). Results are expressed as mean±standard error with *p<0.05, ***p<0.001, when considering all columns versus control in (**B**) and in (**A**) *p<0.05 OS-TA versus OS. Four animals were used in each study.

In primary culture rat RPE cells, OS phagocytosis significantly enhanced COX-2 expression (Figure 11A; ***p<0,001, OS versus control). TA decreased significantly the expression of COX-2 under basal condition (Figure 11A; *p<0.05 versus control) and also significantly reduced the OS phagocytosis-induced COX-2 upregulation (*p<0.05 OS-TA versus OS). Interestingly, while TA also significantly downregulated basal VEGF expression (Figure 11B; *p<0.05 TA versus control), it did not influence OS phagocytosis-induced VEGF upregulation (Figure 11B; ***p<0.001 OS versus control).

## Discussion

Glucocorticoids are known to induce vascular effects. The McKenzie test, based on skin bleaching, has been the basis for the classification of glucocorticoid potency and cutaneous absorption [31]. This test uses the visual quantification of the skin whitening after 8 to 48 h of exposure to different glucocorticoids applied topically and under occlusion. Although this test is widely used, the exact mechanism of skin whitening remains unknown and unexplored. The effects of steroids on growing vessels have been described by Folkman and Ingber [32]. They showed that only a low dose of dexamethasone (0.2 µg/µl) or hydrocortisone (5–6 µg/ml) could induce the regression and prevent the growth of neovessels in the chorioallantoid membrane. High doses, over 10 µg/ml, were toxic. Folkman experiments with chemically modified steroids demonstrated that the vascular effect was not related to the corticosteroid activity but to their effect on the basal membrane of growing vessels. Further studies show that steroids inhibit human endothelial cells proliferation [33], or promote human dermal microvascular cells [34], these effects depending on the type of steroid, the doses and the duration of exposure [35].

In many ocular models of angiogenesis and of human pathologies, the growth of neovessels takes place in a complex wound healing process, where inflammation and angiogenesis are closely interlinked [36]. Therefore, the effects of glucocorticoids result from both a strong anti-inflammatory activity and a direct but unclear effect on vessels.

In vitro experiments performed in our study allowed us to analyze the direct effect of TA on BRECs. We have demonstrated that the effects of TA depend on both the TA solubility and the duration of exposure. At 1 mg/ml, TA did not dissolve completely in ethanol 1%, and the observed necrosis was due to the direct contact of crystals with the cultured cells as previously observed with RPE and rat Müller glial (RMG) cells [25]. When confluent cells were exposed to 0.1 mg/ml TA-E, a clear decrease in the number of living cells was observed, which resulted from noncaspase-dependent apoptotic pathways. Similarly Hartnett et al. found that TA induced a dose-dependent toxic effect on human retinal microvascular endothelial cells, without any activation of the caspase-3 [15], suggesting that noncaspase-dependent apoptotic pathways could be activated.

In our experiments, LEI/L-DNase II was associated with apoptosis of BRECs after 72 h of TA exposure. This is an important result because this enzyme produces 3′P ends [37] in DNA, a break that is not labeled by terminal transferase. This explains why theses cells showing apoptotic morphology and Annexin V staining remain TUNEL negatives. Moreover, as this is the regular technique used to evaluate the induction of cell death in a tissue, many dying cells remain unrevealed. In addition to LEI/L-DNase II, AIF is also nuclearized, suggesting that it is involved in the death of cells after 5 days of TA exposure. So that, multiple caspase-independent mechanisms may mediate this cell death [38]. Despite an abundant literature on the anti-angiogenic activity of steroids, this is to our knowledge, the first study to analyze the mechanisms of TA-induced endothelial cell death. At low dose (1 µM), TA did not influence BREC proliferation, while a moderate effect on cell proliferation was observed at the dose of 0.1 mg/ml, confirming the dose-dependence of endothelial cell proliferation..

The more striking observation was that after intravitreous injection, TA also induced vascular changes, mostly at the level of the choroids. We could not measure precisely in vivo whether TA induced any changes in retinal vessel diameter as demonstrated in patients [39], but our histological analysis seemed to confirm a potential effect of TA on superficial retinal capillary diameter. However, no striking effects on the retinal vessel structure were observed. Moreover, using both semithin and ultrathin analysis and choroidal casts, we could observe a reduction in the choroidal thickness, a rarefaction of cells in the choroids, and a rarefaction of choriocapillaries with increased spaces between the capillaries. At this stage, it is not possible to determine whether the increased space between capillaries results from a loss of capillaries or a vasoconstriction of the vessels. Further studies are undertaken to evaluate potential toxic effect of TA on resting versus proliferating choroidal neovessels.

Interestingly, endothelial cell damage has been reported in veins of rabbits treated with 4 mg/kg methylprednisolone acetate for four weeks. They show ultrastructural alterations of endothelial cells and pericytes [40] suggesting that in vivo steroids may be toxic for the vasculature. This observation is in agreement with the superficial skin ecchymoses known to occur after long-term corticotherapy [41]. Finally, Blaha et al. described the occurrence of profound choroidal hypoperfusion in patients treated with combined photodynamic therapy and intravitreous injections of triamcinolone [42]. It remains to be determined if this complication is related to the direct potential effect of TA.

The relative selective effect of TA on the choriocapillaries as compared to the retinal vasculature in vivo may result from different factors. First, the choriocapillary endothelium is fenestrated and much less protected than retinal capillaries, which are surrounded by pericytes, astrocytes, and retinal Müller glial cells. So that, the choriocapillary endothelium may be more exposed to TA toxicity than the retinal endothelium. Aside from a direct toxic effect, TA could exert an indirect effect through the downregulation of COX2 in RPE cells. In a previous study, we indeed demonstrated that CD36^ −/−^ mice that produce reduced COX-2 levels in RPE cells develop a progressive choriocapillary involution. The activation of COX-2, induced by OS phagocytosis could be important in the homeostasis of choriocapillaries [26]. Interestingly, in our experiments, while TA in vitro prevented the upregulation of COX2 under OS phagocytosis, it did not affect VEGF upregulation. Whether other pro angiogenic factors are downregulated downstream COX2 in RPE cells will be investigated.

Other pro angiogenic factors could be downregulated downstream of COX-2 in RPE cells. Moreover, when angiogenesis develops in the choriocapillaries, as in age-related macular degeneration, the anti-inflammatory and anti-proliferative effects of TA may be involved in its antivascular effects [13]. These issues will be matters for future investigation.

In conclusion, this is the first study to demonstrate that glucocorticoids induce direct retinal endothelial cells toxicity through noncaspase apoptotic pathways involving LEI/ L-DNase II and AIF. While long-term exposure to TA did not induce any detectable toxicity for retinal vessels in vivo, it did lead to choriocapillary rarefaction. Taking into account that those changes may occur without any inflammatory reaction, in the long term, they may be overlooked in clinical practice and induce deleterious consequences on visual acuity. However, because Lewis rats are albinos, and because the rat retina differs from the human retina, no direct extrapolation can be made regarding toxicity in patients. This study is an indication that triamcinolone may exert some effects on the choroidal vessels and that this parameter should be monitored particularly in patients with preexisting chorioretinal pathologies. The effects of TA injected in the suprachoroidal space of pigmented rats are currently under investigation to complete the present study. Further studies should be undertaken to characterize precisely whether choriocapillary changes occur in patients treated with repeated intravitreous injections of TA.
